# Engagement and outcomes of marginalised young people in an early intervention youth alcohol and other drug program: The Street Universities model

**DOI:** 10.1371/journal.pone.0286025

**Published:** 2023-05-18

**Authors:** Theresa Caruana, Limin Mao, Rebecca M. Gray, Joanne Bryant

**Affiliations:** Centre for Social Research in Health, University of New South Wales, Sydney, NSW, Australia; University of the Witwatersrand, SOUTH AFRICA

## Abstract

**Background:**

Early intervention alcohol and drug (AOD) programs for disadvantaged young people have the potential to substantially decrease the need for future intervention, however there is little research about how young people use these programs or the substance use and other outcomes of such programs. This paper uses data from an Australian AOD early intervention program, The Street Universities, to: describe young people’s participation; examine changes in substance use and wellbeing over 90 days; and assess which young people are most positively impacted

**Methods:**

Data come from a prospective study of new attendees, measuring retention in and attendance patterns in an ‘engagement’ program focussed on arts and lifestyle activities (n = 95), and a routine service dataset collected from seven years of therapeutic intervention (n = 3,893), measuring substance dependence (SDS), psychological distress (K10) and quality of life (EQoL)

**Results:**

Analysis reveals that young people were retained in the program at high proportion (63% at six months) and more than half of these returned at a frequency of weekly or more often. Young people participating in the therapeutic component of the program reported significant improvements in all key wellbeing indicators with SDS, K10 and EQoL scores significantly improving (at *p* < .001). These improvements occurred rapidly, within the first 30 days, and were maintained over the 90 day study period. Moreover, young people with the highest SDS and K10 scores and lowest quality of life at baseline experienced the most positive changes.

**Conclusion:**

Aligning engagement program with therapeutic intervention can provide comprehensive support to disadvantaged young people, producing substantial improvements in AOD use, distress and wellbeing.

## Introduction

For young people with multiple disadvantages, youth services can be one of the few sources of social and health support [1, 2], and youth alcohol and other drug (AOD) programs form part of this support. Youth services can provide environments of safety, where youth workers with nonjudgmental approaches can form therapeutic relationships with young people based on trust [2–7]. These supportive environments and relationships help young people to contend with the conditions of their lives, including poverty, criminal histories, substance use, and poor mental health, among other factors [for example, 8–13]. These approaches require effective service provision in two regards: to engage disadvantaged young people in a service setting over the long-term, and to address the unique health and social needs of each young person, including their substance use.

In Australia, for young people with substance use problems, there are a range of health and social services to help them manage their substance use. These include residential and non-residential treatment, detoxification, substitution treatment and a small number of early intervention outreach programs [14]. Not all young people who use these services are disadvantaged or have complex needs, however those who experience poverty, have mental health problems and/or criminal justice involvement are significantly over-represented in the Australian system [15]. Additionally, it is noted that disadvantaged young people are more difficult to retain in AOD services because of their complex needs and difficulty trusting service providers [1, 16].

Early intervention can reduce the need for significant future intervention [17, 18]. Studies show that early intervention can reduce substance use as well as reduce criminal behaviour and foster retention in education [19–21]. As these studies reveal, the evidence about outreach youth AOD tends to focus on outcomes and is less focussed on the particular service approaches used that support positive outcomes [22]. Successful strategies are thought to include having an accessible and accepting service that facilitates peer connections, where youth workers responsively offer psychosocial screening, counselling, and referrals to meet further support needs [5, 7, 22, 23]. Referrals to other supportive services that help young people to develop competencies and positive expectations are also thought to be important [4, 24–26]. There is growing interest in the AOD sector in offering services using a person-centred framework that seeks to support clients to make informed decisions about their care in ways that pay attention to individual abilities, preferences and goals [27, 28]. Yet, as Fomiatti et al. [22] identify, there are very few studies examining the effectiveness of these strategies in outreach youth AOD services. This is partly because such services target a very diverse range of young people––those at mainstream and alternative schools [29], those involved in juvenile justice [20], sex workers [30] and street-involved young people [31]––meaning that engagement strategies are targeted at the specific contexts and needs of these diverse young people.

This paper is one of the first known papers to present data on both engagement/retention patterns and AOD outcomes. It does so using two separate datasets from Australian outreach youth AOD services, The Street Universities, to present detailed information about a cohort of young people who attend the service and their patterns of participation, together with the AOD outcomes of a cohort of clients using the treatment component of the program by engaging in counselling, case management, or educational interventions. Specifically, the paper:

1. describes patterns of retention and engagement among new attendees of the program (including attendance patterns, activities undertaken while attending, and among those who did not return to the program the main reasons given for not returning);
2. examines changes in substance use, psychological distress and quality of life among clients using clinical services over a 90 day period;
3. explores which groups of young people benefit most in the 90 day period, by examining how changes in substance use, psychological distress and quality of life differ for those who report the poorest scores compared to those with higher scores;
4. examines which factors were independently associated with improvements in substance use over a 90 day period.

### The Street Universities program

The Street Universities program runs out of seven locations in Australia, the first established in 2007, and all are purposely located in areas of socioeconomic disadvantage. The service provides a space for young people to socialise away from the street and police attention. Its design has two main components: a comprehensive engagement program aimed to attract and retain young people in the service over the long-term, and a therapeutic program available for those clients who need or want AOD and/or mental health support. While improvements in AOD use and mental health are key outcomes, the program also seeks to positively impact clients’ lives more broadly by offering employment and life skill development, opportunities for volunteering, leadership, and peer mentoring, and bridging programs to further education. These opportunities are offered both within the service and by connecting young people to local businesses and community mentors.

The service operates with guiding principles that it is low-threshold, strengths-based, person-centred, and youth-led. These principles means that barriers to the program are minimised and young people can attend without the need to register or share personal information if they choose. All programs are offered using strengths-based values and practices that foreground self-determination and celebrate the capacities of young people, and therapeutic care is offered using person-centred principles that support clients to make informed decisions about their own care noting their individual abilities and goals. And finally, the service is youth-led, whereby service managers monitor ongoing program development with advice from a youth advisory group.

The engagement component of the program includes, at a basic level, offering safe and fun spaces for socialising or using computers and telephones, but for those who are interested there are also high-quality activities involving arts, dance, music, and life skills training. Material support such as food hampers are also available [32]. The therapeutic program uses a naturalistic person-centred approach whereby AOD and mental health intervention and referral is offered to those who need or want it, as often as they wish and in the format they desire. Inherent in the program design is that the engagement program is targeted at the wide diversity of disadvantaged youth living in the local area, but the therapeutic program is aimed at the sub-group that may need or want AOD and/or mental health support. Some Street Universities sites also operate an outreach program whereby youth workers bring skills training and therapeutic support to young people in their own settings.

## Methods

### Study design

Two sources of data were used, each drawing on a longitudinal design:

1. *New attendee retention and engagement dataset*, which was a prospective cohort study of all new attendees of The Street Universities’ program over a six-month period in 2018. This data was utilised to explore aspects of client retention and engagement in the overall program.
2. *Routine service clinical intervention dataset*, which was clinical assessment data collected over six years from clients participating in the therapeutic component of the program. This data was utilised to assess client outcomes by analysing changes in substance dependence, psychological distress and quality of life over 90 days.

Ethics approval was obtained from the Human Research Ethics Committee at UNSW (17602) and the Aboriginal Health and Medical Research Council of NSW (1292/17).

### Procedure

#### New attendee retention and engagement dataset

This data was collected of all new entrants aged 14–25 over a six-month period in 2018, as part of a service evaluation. Self-completed surveys were administered by study researchers and service staff at three timepoints: baseline, two and six months (within a 28-day window). Those who returned to the program completed their surveys on site. Those who did not return were sent an SMS link inviting them to complete surveys remotely. Participants received stepped incentives for survey completion ($30AUD at baseline, $40 at two-months, $50 at six-months). Participants were contacted four times before they were considered lost to follow-up. Participants aged 14 or 15 either provided written parental consent or successfully completed a competency assessment administered by researchers or staff, which assessed their capacity and maturity to provide their own consent (following the mature minor’s consent concept used in medical, health and research settings [33]. Participants aged 16 and over provided their own written consent.

#### Routine service clinical intervention dataset

This data was collected as part of routine service activities from the sub-group of clients participating in the therapeutic component of the program by receiving counselling, case management, or educational interventions. In the person-centred model offered by The Street Universities, clients can take up as much or as little intervention as they wish, although assessment of their therapeutic progress are taken at ‘checkpoints’ approximately every 30 days. This means after clients receive their first therapeutic intervention they could continue this daily for the next 30 days until the next ‘checkpoint’ when they are assessed again; or alternatively they could take part in it weekly or less frequently until the next ‘checkpoint’. Thus, assessment of progress happens separately from intervention activities. Staff aim to undertake ‘checkpoints’ every 30 days for those who return to the service within a 10-day window around the ‘checkpoint’. ‘Checkpoint’ data includes demographic information, drug and alcohol use including severity of dependence scale (SDS), and wellbeing indicators including psychological distress (K10) and quality of life (EQoL).

This method of routine data collection means that ‘treatment dosage’ can be different for each client as it is led by them and their needs, therefore the data is not a measure of dosage. It also means that the data is not an adequate representation of retention in the therapeutic program because the ‘checkpoints’ happen separately from therapeutic intervention activities. Thus, failure to recapture clients at 30 day intervals could be for a range of reasons unrelated to their participation in therapy, including that they did not attend the service within the 10-day ‘checkpoint’ window. Relatedly, given the person-led approach to therapy, there is no prescribed endpoint for treatment, thus no data is collected on ‘exit’, and we do not know whether clients who do not appear in the dataset have completed treatment to their satisfaction or if they have not reappeared because they have left the service after returning to school or finding employment. Thus we cannot surmise retention in the therapeutic component of the program from this dataset.

For the purposes of this research, three sites of The Street Universities provided clinical intervention data covering a six-year period (2014 to 2020).

### Participants

For the *new attendee retention and engagement dataset*, follow up data was obtained and linked for 95 participants. The average age at intake was 17 years (with a range of 14 to 24), and 55% were female, 45% male. In the *routine service clinical intervention dataset*, 3,893 clients completed a baseline assessment and were included in the analysis. The average age at baseline was 19 (with a range of 12 to 26), 72% were male, 18% female, with 0.3% non-binary.

### Measures

#### New attendee retention and engagement dataset

Retention and engagement in the overall program was measured using the new attendee dataset. ‘Retention’ was measured by calculating the proportion of new attendees who returned after the first visit, the self-reported frequency at which they returned, the self-reported average amount of time they spent at the service per visit, and among those who did not return, the reasons for not returning. ‘Engagement’ in the program was measured by calculating the proportion of attendees who participated in activities. These included: music, dance and art workshops, cooking workshops, counselling, socialising with friends and staff, taking food packages. For those who did not return to the service, the survey included open-ended questions asking for the main reasons why they did not return.

#### Routine service clinical intervention dataset

Three scales with established psychometric properties indicating valid and reliable use in adult samples [34–36] were administered to young people participating in therapeutic services by service staff. Total scores were computed by the researchers.

*Severity of dependence scale (SDS)*. The Severity of Dependence Scale (SDS) measures frequency and severity of substance dependence symptoms over the past three months. Respondents nominate a primary substance of concern and answer five questions (e.g., ‘Did you wish you could stop?’) using four response options (0 = never or almost never, 3 = always or almost always), that sum to a total score of between 0 (no dependence) to 15 (severe dependence) [SDS; 34]. SDS has high reliability and validity with young people [37]. Intake SDS scores in the routine services dataset showed good internal validity (Cronbach’s alpha = .86).

*Psychological distress scale (K10)*. The Kessler Psychological Distress Scale (K10) measures psychological distress symptoms over the past 30 days. Respondents answer ten questions (e.g., ‘About how often did you feel nervous?’) using five response options (1 = none of the time, 5 = all of the time), that sum to a total score of between 10 to 50 with scores of 10–19 being ‘well’, 20–24 ‘mild’, 25–29 ‘moderate’ and 30 and over ‘severe’ [K10; 35]. The K10 has high reliability and validity with Australian child and adolescent populations [38], and Aboriginal and Torres Strait Islander populations [39, 40]. Intake K10 scores in the routine services dataset showed excellent internal validity (Cronbach’s alpha = .94).

*Quality of life scale (EQoL)*. The EUROHIS-QOL 8-item index (EQoL) [EQoL; 36] measures life satisfaction and quality over the past two weeks. Respondents answer eight questions. Questions were asked that measured perceived life satisfaction and quality on a five-point scale. A total score of between 8 (poorest quality) to 40 (highest quality) was derived. Intake EQoL scores in the routine services dataset showed excellent internal validity (Cronbach’s alpha = .90).

#### Sample characteristics

In the *new attendee retention and engagement dataset*, self-reported demographic information included age, gender, Aboriginal or Torres Strait Islander identity, sexual identity, educational status, employment status, living arrangements, and recent drug or alcohol use. In the *routine service clinical intervention dataset*, self-reported demographic information included age, gender, Aboriginal or Torres Strait Islander identity, country of birth, arrests, main income source, principal drug of concern, referral source and time receiving services in days.

### Data analysis

#### Aim 1: to describe patterns of retention and engagement

Descriptive statistics were applied to the *new attendee retention and engagement dataset*, including calculating proportions, means, standard deviations, medians and interquartile range. Differences between those who returned and those who did not were examined using a chi-square test for categorical data and independent t-tests for continuous data.

#### Aim 2: to examine changes in substance use, psychological distress and quality of life among clients using clinical services over a 90 day period

This analysis used the *routine service clinical intervention dataset*. As a first step, exploratory analyses were conducted using Bonferroni adjusted independent t-tests to examine differences in age and intake scores (SDS, K10, EQoL) between those who only completed an intake assessment and those who provided assessments at two or more timepoints within a treatment episode. Differences by gender and Aboriginal and/or Torres Strait Islander identity were examined using a chi-square test.

Following this, we analysed the data for those who undertook assessments at more than one timepoint following a method detailed by Kelly et al. [41]. We chose this method because of its capacity to provide interpretable findings about the proportion of participants who improved, deteriorated or had no change in SDS, K10, and EQoL over the study period. The method involved calculating the standard error of measurement (SEM) for each outcome (SDS, K10, EQoL). Briefly, scores were only included in the analysis where they matched the following standardised timepoints after intake (1–14 days): 30 day interval (15–44 days), 60 day interval (45–74 days), and 90 day interval (75–104 days). In the case where two or more assessments fell within the same time period, only the first survey scores are utilised. SEM was calculated as:

$$SEM=SD_{intake\;outcome\;score}\;*\sqrt{\left(1\;–\alpha \right)}$$

where α refers to the internal validity finding of the intake scores for each scale, giving the results of 1.4 for SDS, 2.25 for K10, and 1.8 for EQoL. Thus, the criteria for statistical significance required participants to move at least 1 point on the SDS, and 2 points on the K10 and EQoL; intake scores lower than 1, 12, and 10 respectively were omitted. Participants were classified as deteriorated, not changed, or improved according to the SEM criteria. Paired t-tests with a Bonferroni-corrected alpha of 0.006 were conducted to report statistical changes between intake and each timepoint, and Cohen’s d effect sizes indicated the strength of the relationship between each pair. Power analyses conducted using G*Power version 3.1, indicated that the sample sizes were sufficient for detecting small effect sizes for each test (alpha = .05, power = .8).

#### Aim 3: to explore which groups of young people benefit most in the 90 day period

This analysis used the *routine service clinical intervention dataset*. Participant scores were categorised into tertiles for those who reported the lowest, middle and highest scores for SDS, K10 and EQoL at intake, and included all participants with scores recorded at more than one timepoint. Mean scores for each tertile were plotted using line graphs with 30 day interval timepoints (calculated as previously described) on the x-axis and SDS, K10 and EQoL on the y-axis.

#### Aim 4: to examine which factors were independently associated with improvements in substance use over a 90 day period

This analysis used the *routine service clinical intervention dataset*. Multiple linear regression was used to test if the magnitude of difference in SDS score between intake and the last recorded timepoint could be predicted by the following variables: gender (6 self-reported non-binary participants were omitted from analysis), Aboriginal and/or Torres Strait Islander identity, SDS intake tertile group (1 = lowest substance dependence, 2 = middle, 3 = highest substance dependence), and the difference in K10 and EQoL scores between intake and the last recorded timepoint. An analysis of residuals confirmed the assumptions of linearity.

All data analysis was conducted using RStudio 2021.09.0.

## Results

### Aim 1: to describe patterns of retention and engagement

Analysis of the new attendee dataset indicated that The Street Universities were able to attract their target group of disadvantaged young people and retain a considerable proportion at high frequency over a six-month period. Of the n = 95 participants in the new attendee data set, 63% returned at least once during the six-month follow-up period, and more than half of these returned at a frequency of weekly or more often (Table 1). Visits were an average of two hours and twenty minutes. Socialising with friends was the most popular activity, although participants also commonly reported using the service to access computers and to socialise with staff. There were no significant differences between those who returned (n = 60) and those that did not (n = 35) in age and other demographic details, or alcohol and drug use (Table 1). Among those that did not return to the service, the reasons provided for not returning related to being too busy with work, study or family (n = 26, 74%) problems with transport (n = 2, 6%) and not feeling a need to return (n = 7, 20%), therefore were reasons not related specifically to the features of the service.

**Table 1:** Retention and engagement measures and demographic characteristics, new attendee retention and engagement dataset, 2018 (n = 95*)*.

	Continued to attend (*n* = 60)	Ceased to attend (*n* = 35)	χ^2^, t, df*
	*N* (%)	*N* (%)	
***Demographic information***			
Age (mean, SD, range)	17 (2.6, 14–24)	17 (2.7, 14–24)	
Male	26 (43)	17 (49)	
Female	34 (57)	18 (51)	0.67 (1)
Aboriginal/Torres Strait Islander	8 (13)	3 (9)	0.17 (1)
LGBTQ	11 (18)	3 (9)	0.36 (1)
Recent homelessness	12 (20)	7 (20)	0.6 (1)
Education/employment			
At school	26 (43)	17 (49)	0.67 (1)
In tertiary study	14 (23)	5 (14)	0.43 (1)
Employed	8 (13)	10 (29)	0.10 (1)
***Drug and alcohol use***			
Reported any drug use of concern	28 (47)	10 (28)	0.28 (1)
Drank alcohol in past 4 weeks	37 (62)	23 (66)	0.83 (1)
Used cannabis in past 4 weeks	24 (40)	14 (40)	0.58 (1)
***Patterns of program participation***			
*Retention and engagement*			
Attended at least once	60 (63)		
Attended once a month or less	14 (25)		
Attended 2–3 times per month	12 (21)		
Attended once a week or more	31 (54)		
Average amount of time spent at service per visit (median, IQR)	2.3 (2–3)		
***Participation in program elements***			
Socialise with friends	48 (89)		
Socialise with staff	24 (40)		
*Practical needs and life skills*			
Use computers	29 (48)		
Taken food package	10 (17)		
Cooking workshops	8 (13)		
*Arts workshops*			
Music workshops	15 (25)		
Dance workshops	15 (25)		
Graffiti art workshops	6 (10)		
*Therapeutic counselling*			
Alcohol or drug counselling	5 (8)		
Mental health counselling	8 (13)		

Note.

*estimated using Fisher’s Exact test for categorical data and independent t-test for continuous data; all tests were non-significant (p >.05)

### Aim 2: changes in substance use, psychological distress and quality of life among clients using clinical services over a 90 day period

In the routine services dataset, cannabis was the most prevalent principal drug of concern, followed by amphetamines and alcohol. Most young people were referred to The Street Universities from community services (including local health services, youth justice, or AOD services) and through court diversionary processes. Of those that had ever been arrested, the median arrest frequency was once (*M* = 2.65, *SD* = 5.84, range 1 to 90). Table 2 presents the demographics of this cohort.

**Table 2:** Intake demographic characteristics and time in service, routine service clinical intervention dataset, 2014 to 2020 (n = 3893).

*Characteristic*	*N* (%)
*Demographic information*	
Male	2792 (72)
Female	1091 (28)
Non-binary	11 (0.3)
Aboriginal and/or Torres Strait Islander	675 (17)
Born in Australia	3407 (88)
*Main income sources*	
Employed	834 (21)
Income dependent on family/others	1111 (29)
Receive government allowance/pension	1572 (40)
*Principal drug of concern at intake*	
Cannabis	1660 (43)
Amphetamines	955 (25)
Alcohol	812 (21)
Nicotine	182 (5)
Benzodiazepines	65 (2)
Opioids	48 (1)
Cocaine	34 (0.9)
*Housing status*	
Homeless or in supported accommodation	169 (5)
*Forensic history*	
Ever arrested	776 (19)
*Referral source*	
Community services (including local health services, youth justice, AOD services)	2261 (58)
Court diversion	783 (20)
Hospital	449 (12)
Self	231 (6)
Family/friend	103 (3)
AOD service	51 (1)

Fifty-two percent of clients completed only the first ‘checkpoint’ assessment (n = 2025) and were not recaptured in the data. No significant differences were found in age or intake SDS, K10 and EQoL scores or in gender (*X*^2^(2, *N* = 3893) = 3.0, *p* = .220, φ = 0.03) and Aboriginal and/or Torres Strait Islander identity (*X*^2^(1, *N* = 3893) = 2.6, *p* = .268, φ = 0.02) between those who only completed a baseline assessment and those who completed further assessments. Mean SDS, K10 and EQoL scores significantly improved (at *p* < .0006) from baseline to each timepoint, with most experiencing improvements on one or more measures. The greatest effect sizes and proportions reporting improvement were in reference to reductions in psychological distress (Table 3). Overall, 50% of young people who were recaptured in the service data reduced their substance dependence, 52% experienced improved quality of life, and 71% experienced reduced distress within 90 days of service.

**Table 3:** Statistically significant changes between assessments for substance dependence (SDS), psychological distress (K10), and quality of life (EQoL) at four standardised timepoints, routine service clinical intervention dataset 2014 to 2020 (n = 3893).

									*Standard error of measurement*		
Timepoints	*N*	*M*	*SD*	95% CI lower	95% CI upper	*t*	*df*	*ES*	Deteriorate	No change	Improve
*Substance dependence (SDS)*											
Intake (ref)	3728	4.16	3.74	4.04	4.28						
30 days	887	3.10	3.11	2.84	3.25	9.94*	886	.31	24.1%	28.1%	47.8%
60 days	490	3.05	3.15	2.77	3.33	6.69*	489	.30	24.3%	28.0%	47.8%
90 days	253	2.94	2.95	2.58	3.31	6.14*	252	.33	23.3%	27.7%	49.0%
*Psychological distress (K10)*											
Intake (ref)	3331	23.28	8.64	22.98	23.57						
30 days	769	19.89	7.82	19.34	20.45	15.37*	768	.40	17.3%	23.0%	59.7%
60 days	430	19.08	7.68	18.35	19.81	12.34*	429	.49	15.8%	17.9%	66.3%
90 days	229	19.73	7.89	18.70	20.76	10.40*	228	.41	17.9%	11.4%	70.7%
*Quality of life (EQoL)*											
Intake (ref)	3892	28.03	5.70	27.85	28.21						
30 days	891	29.41	5.34	29.05	29.76	-7.89*	890	.24	24.6%	33.7%	41.8%
60 days	497	29.64	5.45	29.16	30.12	-7.95*	496	.28	23.3%	27.8%	48.9%
90 days	256	29.54	5.07	28.91	30.16	-6.64*	255	.27	21.1%	27.3%	51.6%

Note. *p < .006 (two-tailed, Bonferroni adjusted alpha). CI, confidence interval; df, degrees of freedom; ES, effect size (Cohen’s d); M, mean; ref, reference. Includes only those young people that completed surveys within the following time periods: 30 days from intake (greater than 14 and less than 45 days), 60 days from intake (greater than 44 days and less than 75 days), and 90 days from intake (greater than 74 days and less than 105 days). In the case where two or more surveys fell within the same time period, only the first survey scores are utilised. Young people with SDS intake scores of 1 or less, K10 intake scores of 12 or less, or EQoL intake scores of 10 or less were unable to be utilised in the SEM calculations and were omitted from analysis. M, SD, and 95%CI include all scores at each timepoint and do not represent t-test baseline scores (which vary according to participants included at each timepoint).

### Aim 3: which groups of young people benefit most in the 90 day period

**Fig 1:**
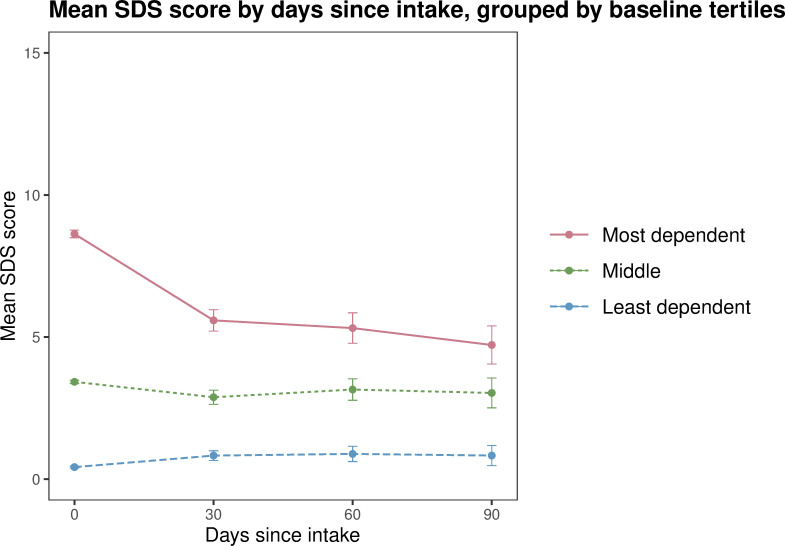
Mean substance dependence (SDS) score by days since intake, grouped by intake tertiles (from least to most dependent), routine service clinical intervention dataset 2014 to 2020 (n = 3728). Note: At 30 days since intake n = 888, at 60 days n = 491, at 90 days n = 255. Error bars indicate 95% confidence intervals.

**Fig 2:**
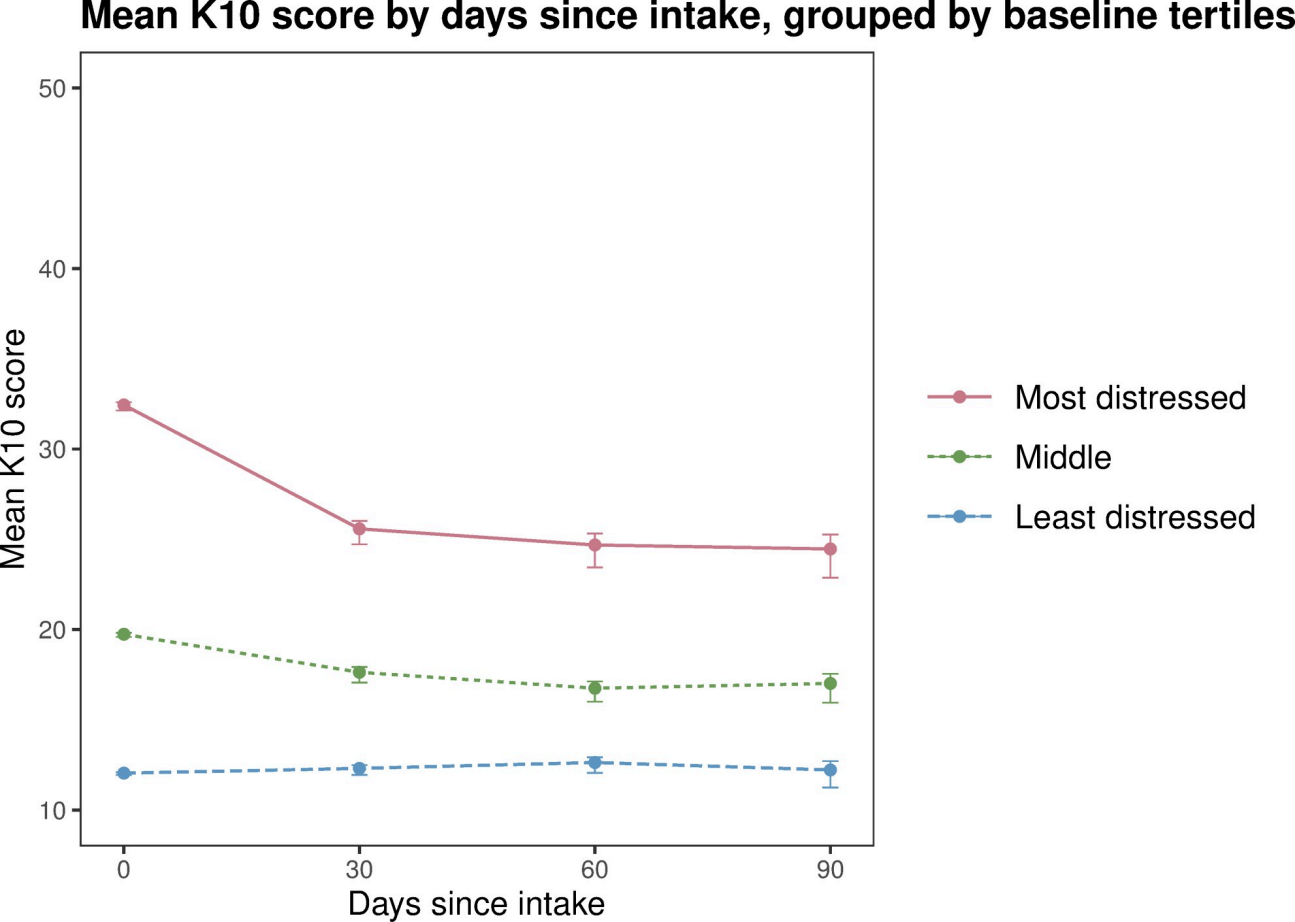
Mean psychological distress (K10) score by days since intake, grouped by intake tertiles (from least to most distressed), routine service clinical intervention dataset 2014 to 2020 (n = 3893). Note: At 30 days since intake n = 891, at 60 days n = 497, at 90 days n = 256. Error bars indicate 95% confidence intervals.

**Fig 3:**
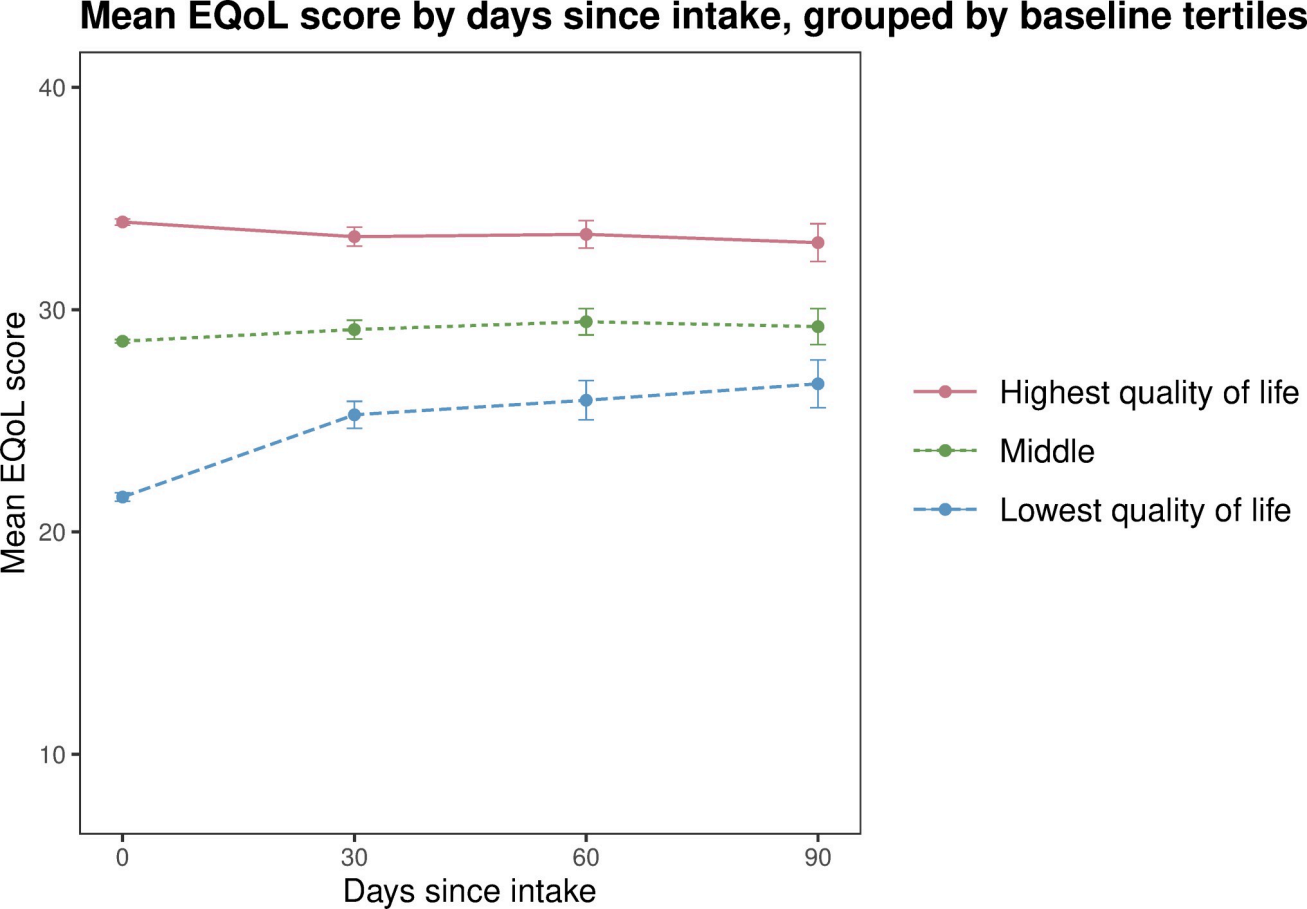
Mean quality of life (EQoL) score by days since intake, grouped by intake tertiles (from lowest to highest quality of life) routine service clinical intervention dataset 2014 to 2020 (n = 3893). Note: At 30 days since intake n = 891, at 60 days n = 497, at 90 days n = 256. Error bars indicate 95% confidence intervals.

The analysis by tertile group indicated that the young people with the highest psychological distress, highest substance dependence and lowest quality of life on baseline experienced the most positive changes. These improvements occurred rapidly (within the first 30 days) and were maintained over the 90 day study period. For example, participants who reported the highest substance dependence at baseline had the greatest mean improvement over the 90 day reporting period (from 8.63 to 4.72, a 45% decrease), with the sharpest decrease occurring between intake and 30 days (to 5.59) (Fig 1). Those with the highest levels of psychological distress at intake also had the greatest mean improvements (from 32.4 to 24.5, a 24% decrease), with the sharpest decrease occurring between intake and 30 days (to 25.6) (Fig 2), indicating that this group of participants moved from the range of ‘severe’ distress to ‘moderate’ over the 90 day reporting period. Quality of life scores followed a similar trajectory of improvement (Fig 3). Notably, minimal changes were observed in the mid and low tertiles, meaning the intervention had limited impact on these participants.

### Aim 4: Factors independently associated with improvements in substance use over a 90 day period

The regression results predicted overall improvements in SDS scores over time, with reductions in substance dependence associated with increased quality of life and decreased psychological distress scores (Table 4). Over a third of the variance in SDS score difference from intake to the last ‘checkpoint’ was explained by K10 score difference, EQoL score difference, gender, and intake K10 tertile group, suggesting that these indicators track together with improvements in one being related to improvements in others. Improvements were greatest for females and for those young people that recorded greater levels of substance dependence at intake.

**Table 4:** Regression results using the difference between first and last substance dependence scale (SDS) scores as the criterion (n = 1648; reference categories: male, non-Aboriginal and/or Torres Strait Islander identity, SDS Tertile 2), routine service clinical intervention dataset 2014 to 2020.

Predictor	*b*	*b* 95% CI [LL, UL]	*beta*	*beta* 95% CI [LL, UL]	*sr* ^*2*^	*sr*^*2*^ 95% CI [LL, UL]	*r*	Fit
(Intercept)	0.10	[-0.15, 0.36]						
Female	-0.48**	[-0.78, -0.18]	-0.06	[-0.10, -0.02]	.00	[-.00, .01]	-.01	
Aboriginal and/or Torres Strait Islander identity	0.23	[-0.12, 0.59]	0.03	[-0.01, 0.06]	.00	[-.00, .00]	-.01	
SDS Tertile 1	-1.23**	[-1.55, -0.91]	-0.17	[-0.21, -0.12]	.02	[.01, .03]	-.38**	
SDS Tertile 3	2.56**	[2.22, 2.89]	0.35	[0.30, 0.39]	.09	[.06, .11]	.50**	
K10 score difference	0.12**	[0.10, 0.14]	0.27	[0.22, 0.31]	.05	[.03, .07]	.41**	
EQoL score difference	-0.06**	[-0.08, -0.03]	-0.09	[-0.14, -0.05]	.01	[.00, .01]	-.30**	
								*R*^*2*^ = .372**
								95%CI [0.34,0.40]

Note.

*p < .05.

**p < .01. K10, psychological distress scale; EQoL, quality of life scale. A significant *b*-weight indicates the beta-weight and semi-partial correlation are also significant. *b* represents unstandardised regression weights. *beta* indicates the standardised regression weights. *sr*^*2*^ represents the semi-partial correlation squared. *r* represents the zero-order correlation. *LL* and *UL* indicate the lower and upper limits of a confidence interval, respectively. SDS Tertile 1 represents the third of participants that self-reported the lowest substance dependency at intake, and SDS Tertile 3 represents the third of participants that self-reported the highest substance dependence at intake, with SDS Tertile 2 representing the third of participants between these.

## Discussion

This research indicates that outreach youth AOD early intervention programs can retain disadvantaged young people in a service environment and can contribute to significant and rapid improvements in substance use and related wellbeing. For young people that received The Street Universities’ therapeutic services for more than 30 days, indicators of substance dependence, psychological distress, and quality of life all significantly improved. Gains were greatest in the first month of therapeutic counselling, and positive impacts were greatest for those that had relatively lower self-reported wellbeing at intake. Young people that had higher self-reported substance use at therapeutic intake improved more than those with lower levels of substance use, and this pattern was repeated in ratings of psychological distress and quality of life suggesting that these improvements track together. Overall, these findings demonstrate that a low-threshold, strengths-based engagement program can be successful in retaining a considerable proportion of disadvantaged young people over a 90 day period, and that person-centred therapeutic components can be integrated into such programs to produce substantial improvements in AOD use, distress and wellbeing for the sub-group of young clients that are most in need.

Street Universities appear to reach the young people they aim to reach––those who are disengaged from school and work, engaged in substance use, and experiencing moderate to high levels of distress and poor quality of life. A key feature of outreach youth AOD programs is their ability to attract and retain young people [22, 42]. Our data show that high rates of retention can be achieved among young people with high needs creating more opportunity for therapeutic intervention and referral. The Street Universities is a unique program because it is aimed at disadvantaged young people, only some of whom may report AOD problems, which is unlike other early intervention programs described in the literature that tend to purposely exclude young people without obvious AOD problems. Because of this there is no clear comparison regarding retention rates. However, an outreach youth AOD service in New Zealand with a similar emphasis on engagement and voluntary participation in treatment [43] managed to retain 39.9% of clients over four treatment sessions. The Street Universities retention of over 60% in the overall program indicates the strong relevance of the engagement program to young people.

The routine services data revealed that The Street Universities’ therapeutic program can achieve rapid improvements in multiple wellbeing domains, and that these can be sustained over time. Moreover, and consistent with other studies, young people that started with poorer wellbeing indicators improved more rapidly and at a greater magnitude than those with better indicators, as found in some other studies [12, 44]. We also found that the demographic profile in the new attendee and routine service datasets was somewhat different with, for example, the routine service data showing a higher proportion of young men. This suggests that the two programs (engagement and therapeutic) can pick up different groups of young people, but the sub-group that ends up in therapy is slightly older and aligns with the profile other Australian AOD datasets in reporting a high proportion of men [41].

This paper presents data on both engagement in an outreach youth AOD service and therapeutic outcomes, information that is largely missing in the existing literature [22]. Although we cannot link our data about overall program participation and therapeutic outcomes, we do show that the engagement program of The Street Universities is strong, and that therapeutic interventions offered in this setting can achieve significant improvements. This suggests the importance of aligning engagement practices and therapeutic interventions since high quality engagement can prolong the opportunity for therapeutic intervention to work in positive ways. The findings show that improvements in substance dependence track together with improvements in distress and quality of life. This reflects findings from other research that programs that aim to address multiple risk factors (mental health and AOD) simultaneously were more effective than those that targeted only one (e.g., AOD) [45]. This suggests that youth services should seek to intervene on multiple indicators by providing holistic and person-centred supports, since improvements in one area may create improvements in other areas too [41, 45]. In demonstrating the effectiveness of a youth service approach that targets multiple aspects of wellbeing and is responsive to young people’s interests and the social and material challenges they face, it aligns with other research that recommends these types of approaches [22, 42, 46].

This study also offers useful insight for how youth services can monitor impact. Evaluating improvements among youth service clients is difficult due to the diversity of young people’s backgrounds and situations, the diversity and intensity of services they may be receiving, their level of service participation [23, 46, 47], and because adolescent difficulties evolve and change with time [48]. Data collection can be time-consuming for staff and difficult to undertake (especially in opportunistic encounters), and young people may not want to participate [49]. As such, outcome evaluation studies of youth interventions are difficult to achieve [22, 46] and designs that include comparison groups are an added challenge. The routine service data collected at The Street Universities demonstrated how implementing a system to regularly collect information can be useful. Moving forward, the findings of this paper suggest the necessity of integrating service data concerning enrolment, monitoring retention and participation in engagement activities, and directly linking these with outcome assessments to demonstrate a clear intervention pathway.

Finally, while reducing the prevalence of substance use and other potentially harmful practices may improve young people’s wellbeing, these improvements may be difficult to sustain where the social conditions that give rise to these harms remain [22, 50]. Youth services that target disadvantaged young people play an important bridging role in providing relevant supports and opportunities to build self-efficacy in navigating socioeconomic difficulties, but it is unrealistic to assume that these services can fully address or resolve problems that are caused by social inequalities.

### Limitations

Our study provides much more detail than previously available about how young people use outreach youth AOD services, and the positive outcomes that are possible for the sub-group that are most in need, but it cannot directly link patterns of participation with AOD outcomes. In addition, we do not have a comparison group to determine whether young people not engaged with the service experienced any changes in SDS, psychological distress and EQoL over the same time period. Finally, the procedure for collecting routine service data means that we cannot use this data to assess retention in the therapeutic program. That is because the 10-day window for ‘checkpoint’ assessments could have missed clients who did not attend in this this time, and that information about why clients did not return is not available.

In relation to the measures used, evidence for valid use of EQoL or for SDS scales across multiple substances in Aboriginal and Torres Strait Islander or adolescent populations is limited. In relation to data analysis, the use of SEM as a measure of statistically significant change may not reflect clinically meaningful change, and it is not known if improvements are maintained beyond 90 days or after service cessation. Finally, regarding our tertile analysis, grouping participants according to the severity of presenting issues may have limited inferential utility, as category cut-points are determined from a continuous dataset, however this approach does offer increased practical interpretability.

## Conclusions

This research provides an example of an outreach youth AOD service with high retention in engagement programs and an impressive capacity to create positive therapeutic impact for the sub-group of disadvantaged young people that are most in need. Engagement and treatment modalities can work together over the long term to provide safe spaces, social supports and, when needed, therapeutic interventions, so to enable disadvantaged young people to positively manage their substance use in an environment that feels safe and encourages self-realisation and empowerment.
